# A Resilient Health System in Response to Coronavirus Disease 2019: Experiences of Turkey

**DOI:** 10.3389/fpubh.2020.577021

**Published:** 2021-01-07

**Authors:** Bekir Keskinkiliç, Irshad Shaikh, Ahmet Tekin, Pavel Ursu, Adil Mardinoglu, Emine Alp Mese

**Affiliations:** ^1^Ministry of Health (Turkey), Ankara, Turkey; ^2^World Health Organization (Turkey), Ankara, Turkey; ^3^World Health Organization (Switzerland), Geneva, Switzerland

**Keywords:** SARS-CoV-2, management - healthcare, pandemic (COVID-19), public policies, health system - organization and administration

## Abstract

Turkey's response experience thus far with the severe acute respiratory syndrome coronavirus-2 (SARS-CoV-2) pandemic affords the globe and the region a unique opportunity for and distinctive insights into combating this novel virus. The country's pandemic response, having one of the lowest case fatality ratio (2.8%; 52.5 infections/million population), particularly among the elderly (the high-risk group), rising to the occasion to shoulder its long-standing role in global solidarity and humanitarian support by providing personal protective equipment (globally scarce) to many countries in their desperate time of fight against the pandemic while also meeting its own critical domestic needs, stands out. This paper aims to highlight key decisions, actions, and partnerships behind Turkey's successful fight against the SARS-CoV-2 pandemic that have enabled the country to turn the corner, as well as the components of its success story.

## Introduction

Turkey's response to and experience thus far with the severe acute respiratory syndrome coronavirus-2 (SARS-CoV-2) pandemic affords the world and the region a unique opportunity for and distinctive insights into combating this novel virus. On the one hand, Turkey has one of the lowest case fatality ratios (2.8%; 52.5 infections/million population), particularly among the elderly aged 65 years and older (the high-risk group). It has also risen to the occasion and shouldered its role as a long-standing propagator of global solidarity and provider of humanitarian support. Turkey's success in scaling up local manufacturing of personal protective equipment (PPE), a globally scarce commodity, and dispatching supplies on humanitarian grounds to many countries in their time of desperate need to fight against the pandemic, while still able to meet critical domestic needs, offers key lessons in manufacturing and adjusting supply chains. This paper aims to highlight key policies and practices and partnerships behind Turkey's effective and successful fight against the SARS-CoV-2 pandemic until the end of May 2020. These have enabled the country to significantly lower the case load and to expand upon the elements behind this success.

## Prevention and Preparedness

### Health Systems Reforms

Turkey, though geographically located between Europe and Asia, is also a Mediterranean country with Mediterranean heritage and culture. Close contact and conviviality is part of its long-standing and rich tradition. Spending time together and congregating during social events and hugging and cheek-to-cheek contact are very common greeting gestures in daily life. Such physical contact-based cultural factors become particularly important when considering infection prevention and control measures at the population level for an affliction that is inherently spread by droplet and close contact.

Turkey has been implementing a health reform initiative called the Health Transformation Program since 2002 (1). This program has covered and changed nearly all building blocks of health systems in Turkey—from governance to health financing to health service delivery, with heavy investments in health infrastructure, redefining the roles of all key relevant stakeholders for the better (2).

Three key macrolevel features of this health system transformation that have played critical enabling roles during the pandemic are as follows:

1. Strengthening of primary health care (PHC). With accessibility and equity as foundational principals, staff in PHC facilities links peoples and communities through a network of nearly 8,000 hubs with 25,000 family medicine units. Each serves, free of charge, a surrounding catchment unit of 3,000 persons and thus traversing the geographical and social extent of the country. Every person in this 3,000 catchment population unit thus has an assigned family physician in charge of their health, facilitated by electronic health records for each, including street address records of all. This comprehensive PHC network with improved access to and an up-to-date health and geographical information on each person made community outreach and engagement for the SARS-CoV-2 response efficient, effective, and timely, from risk communication to testing to contact tracing.
2. Turkey built large “healthy cities” harnessing a public–private partnerships model that boosted its health infrastructure manifold, especially intensive care bed capacity (3),^1^ with some hospitals specifically equipped with negative pressure rooms—assets and capacities that proved decisive in saving lives among those severely ill with SARS-CoV-2 without seriously straining critical care systems and capacities.
3. The population was extensively covered with a reliable information technology (IT) infrastructure that enabled and supported critical response elements. These ranged from timely reporting of surveillance and early warning to telemedicine for the elderly and those with chronic diseases, as well as those with mental health problems and home/facility-bound persons. It also connected those who were “healthy but worried” with a healthcare provider, precluding crowding of health facilities and possibly excessive healthcare worker (HCW) infections.

Before the pandemic, Turkey had one of the most comprehensive Universal Health Coverage schemes [accessible by 99% of all inhabitants including over 3.6 million Syrians seeking refuge in Turkey—Syrians under Temporary Protection (SuTP)] (4).

### Strong Culture of Health Emergencies and Disaster Management

Supported by Health Transformation Program, the country also has a long-standing strong and resilient health system, tested and retested by many natural and man-made disasters and emergencies. A WHO publication of 2011 entitled “Assessment of Health Systems' Crisis Preparedness: Turkey” concluded that “With its broad experience in disaster situations and its advanced disaster and emergency management system, Turkey could play a leading role in training and research related to disaster risk reduction at global level” (5). It is this realization and appreciation of Turkey's expertise in health emergencies and disaster management that has made it appropriate for the WHO Regional Office for Europe to house its new regional center of excellence on Preparedness for Humanitarian and Health Emergencies in Istanbul. This center is part of a system of the WHO Regional Office for Europe's outposts (centers) also called geographically dispersed offices (GDOs)^2^ (6), with each working on and offering expertise in thematic areas.

### Pandemic Influenza Preparedness

Another supporting WHO initiative has been the implementation of an intersectoral approach-based, multidimensional process entitled the Pandemic Influenza Preparedness (PIP) Framework to help Member States prepare for and to be ready to respond to pandemic influenza.

After the publication of the National Pandemic Influenza Preparedness Plan with a presidential decree, members were selected and assigned to the PIP Scientific Consultancy Board (7). Provincial pandemic plans were prepared by provincial health directorates and the Ministry of Health (MoH) organized a workshop for the evaluation of the developed provincial plans. The MoH also organized training of trainers for PIP implementation and training of healthcare workers and the public.

### WHO and International Health Regulations (IHR, 2005)

After restructuring and re-aligning its position on global health emergencies within the United Nations system, the WHO established the WHO Health Emergencies Programme (WHE) in 2016 (8). The IHR (2005) have also defined the core capacities of a strong health system, built on an all-hazards, intersectoral coordination approaches, to manage any public health emergency. Guided by the IHR (2005), the WHE has been leading and coordinating the coronavirus disease 2019 (COVID-19) pandemic and has been ready to respond to other public health emergencies by strengthening preparedness and readiness capacities at country and subcountry levels.

### Progressive Policies and Historical Measures

Turkey has had a long history of prevention and control of communicable diseases starting from the early Ottoman Empire era with its quarantine regulations. Since the early stages of the foundation of the Republic of Turkey, relevant public health and public safety laws and regulations have been consistently updated, improved, and published on public health and communicable diseases. Because of its historical emphasis and experience and capacity building of its physicians and other health workforce, Turkey has been successful in keeping many global and/or regional outbreaks out of its borders and territories. Generations of Turkish citizens have also inherited/embraced a culture of civic responsibility and embracing rules, regulations, and expert guidance from the State, citizenry attributes that have only enhanced the efficiency and effectiveness of the prevention and containment measures instituted against pandemics at national levels.

The foundation of the current policies of Turkey on outbreaks and pandemics emanates from the notification system established in 2004.^3^ This was followed by the creation of the early warning and response system (EWRS) in 2007 for the surveillance and control of communicable diseases.^4^ In addition, pandemic preparedness plans have also been regularly updated and published.

Turkey's years' long and incremental experience with the prevention of outbreaks accumulated over the years, EWRS system, and continuous learning with updated pandemic preparedness plans have helped prevent and control influenza pandemics and other outbreaks on its territory. Turkey's past experiences with swine flu (H1N1), avian influenza (H5N1), and SARS outbreaks only attest to the effectiveness and efficiency of Turkey's policies (9).

### Health Security: an Intersectoral, All-Hazards Approach

In a globally interconnected world coupled by Turkey's geopolitical importance in the region, the country has increasingly recognized the critical need to comply with its global (IHR, 2005) (10) and regional (EC 1082/2006) obligations (11), showing how national and global health security are intertwined and interdependent. For the past 15 years, a series of projects on strengthening surveillance and control of communicable diseases, strengthening and expansion of EWRS, laboratory sector and linking lab surveillance with disease surveillance, and building field epidemiology training have been implemented in collaboration with the WHO and the European Union (EU). These projects have cemented and expanded the health security capacities of Turkey and helped prioritize health threats including those due to emerging and re-emerging diseases and refined and improved EWRS. The country has also updated technical guidelines in alignment with global and regional standards, and best practices of EU and WHO, on a regular basis.

Under these projects, coordination mechanisms have been strengthened for EWRS between the MoH and other line ministries across relevant sectors, and protocols prepared and signed to cement this interministerial collaboration between and across sectors.

Establishment of a national reference laboratory was also a key component of these projects. Within the scope of the currently ongoing Health Security Project, 4th in the series, a laboratory assessment tool was updated, and a capacity assessment study was completed in 2019 that included on-site evaluations of selected laboratories to monitor and evaluate the application of national standards and compliance therewith at the provincial levels. This strengthened and expanded EWRS and the laboratory sector, but more importantly, strengthened linkages between the two have been instrumental in combating this pandemic.

## Readiness Steps of Turkey: Onset of the SARS-COV-2 Outbreak in the World

On December 31, 2019, the People's Republic of China notified the WHO on atypical pneumonia cases of unknown origin in Wuhan. The WHO published its first report on the outbreak on 5 January 2020. Following the decisions of its IHR Emergency Committee, the WHO declared “public health emergency of international concern” (PHEIC) on 30 January 2020 regarding the outbreak of novel coronavirus. The disease was later named as COVID-19 in February. Due to the rapid increase in the number of cases and affected countries, the WHO declared COVID-19 outbreak a global pandemic on 11 March 2020 (12).

### Activation of EWRS—Emergency Operations Center

Turkey activated its preparedness/contingency plans and began its readiness activities soon after the news of the outbreak of this atypical pneumonia in China. As early as January 6, 2020, the EWRS Emergency Operations Center (EOC) in Ankara, Turkey, was activated and situational monitoring of the outbreak from this novel coronavirus in China started with updates from China and through WHO resources. Starting from early February, with cases increasing, this EOC started to work on a 7/24 basis with technical staff manning key/priority technical areas, namely surveillance, logistics, IHR focal point, and official focal points of other relevant line ministries and stakeholders. This operations center continues to remain operational with a similar configuration as of date.

### Convening of the Coronavirus Scientific Advisory Board

On 10 January 2020, just before the announcement of the first fatality by China, the Ministry of Health convened the Coronavirus Scientific Advisory Board (CSAB), bringing together experts from different medical disciplines. CSAB is composed of 26 members, all senior and high-level specialists and academicians in various relevant fields, e.g., public health and epidemiology, pulmonology, infectious diseases, and clinical microbiology, among others. The CSAB has been a critical technical support body since then and has guided not only MoH leadership and staff but also those from other relevant line ministries and other stakeholders. Though formally convening twice a week, in practice, board members spent most of their times at MoH discussing emerging pandemic-related issues thoroughly in detail and in real time, generating discussions and garnering consensus on critical and emerging issues. One of the important and critical outputs especially at the beginning was the drafting of the National 2019-nCoV Disease Guidelines that set the stage for prevention, mitigation, and containment. CSAB meetings were later moved to a videoconference platform. Realizing the importance of communication, an online messaging platform was formed to ensure a constant communication channel. As the needs grew, additional experts/scientists were added to the board, allowing additional technical subgroups to work on emerging priority areas and concerns.

### Release of the 2019-nCoV Disease Guidelines

The first version of the 2019-nCoV Disease Guidelines was published on the MoH website on 14 January 2020 and served as a dynamic, living document (13). As new information and knowledge trickled in, these guidelines were frequently updated to incorporate new knowledge and emerging evidence. Training of healthcare workers was continually conducted at the provincial levels, in line with the national guideline and to ensure the latest global and regional knowledge trickled down and was shared and used at the provincial/municipal levels, the first line of contact between health staff and the community.

### Development of PCR Diagnostic Test for SARS-CoV-2

Laboratory diagnosis with PCR testing was initiated at the National Microbiology Reference Laboratory; however, it was a time- and resource-intensive effort initially. In particular, a research protocol was initiated to develop and produce a rapid laboratory PCR test kit for domestic use as well as for export to other countries. With the help of the WHO country office, the WHO's Emergency Use Listing was readily secured for the newly developed test which also extended to test the necessary quality cover for national use and for international marketing. At the end of May 2020, there were 115 laboratories strategically spread across the country capable of performing PCR test for SARS-CoV-2 infections.

### Initial Steps to Prevent Importation of Disease

Anticipating a high risk of imported cases, temperature screening with thermal scanners was initiated of passengers arriving on flights originating from infection-reporting countries from 24 January at all involved airports. No symptomatic passenger was admitted on Turkish Airlines' planes at points of departure, and all passengers were asked to fill a Passenger Contact Information to ensure efficient and effective contact tracing if any exposure was later suspected on board. Passengers demonstrating any symptom of disease were quarantined. These passenger screenings were later expanded to include all countries that reported a large number of confirmed cases. Later, all arriving international passengers were subjected to 14 days quarantine at designated places.

### SARS-CoV-2 Referral Hospitals

A total of 563 hospitals with the necessary infrastructure and staff were selected to serve as reference hospitals for COVID-19 cases, and all elective procedures and surgeries were put on hold indefinitely in these hospitals. Hospital admissions were minimized and allowed only through a centralized healthcare appointment system—reachable through a hotline, website, or an online app.

### Travel Restrictions

Turkey canceled all flights from China as early as 3 February, followed by Iran on 23 February. Turkey also temporarily closed its border land crossings with Iran for 4 days to mount field hospitals at eight border land crossings and then reopened land border crossings with necessary health screenings.

### Risk Communication and Infographics

On 29 January, brochures, banners, and posters prepared in Turkish, English, and Arabic were distributed to inform the public, highlighting precautions and actions to stop virus transmission. Starting from February, TV spots and social media communication campaigns were broadcasted widely on the media.

### Controlled Airlifting of Turkish Citizens From Abroad

Turkey also evacuated its citizens stranded in disease-prone areas/countries as international travel shrank. The first flight brought stranded citizens from Wuhan on 31 January and the second from Tehran on 23 February. Airlifting continued from several countries since then, and so far, over 70,000 citizens have been brought back home from across the globe under controlled and risk mitigation strategies. All arriving citizens were subjected to 14 days of quarantine at dedicated locations.

### Activation of Provincial Emergency Operations Centers and Health Protection Boards

Provincial operation centers are activated and provincial health protection boards established under the leadership of governors. They started working to manage the pandemic at the provincial level to guarantee effective management.

### Public Engagement and Risk Communication

Showing leadership from the front, Minister of Health Fahrettin Koca regularly held press conferences, especially after scientific board meetings, to inform the public about the latest developments as related to the management of the pandemic, emerging knowledge, and best practices. Risk communication meetings and events (with social distancing observed) were organized to share information and to get feedback from all relevant stakeholders and other governmental ministries and entities.

### Strategic Prepositioning of Critical Personal Protective Equipment and Therapeutic Agents

In line with recommendations made by the CSAB, the MoH ensured provision of appropriate and adequate amounts of therapeutic regimens such as hydroxychloroquine, the antiviral favipiravir, and other drugs for use in hospitals and PPE for healthcare workers in healthcare settings. Sufficient stockpiles of these critical drugs were ensured before the start of the pandemic in the country.

### Overcoming Challenges to Be Ready for the Pandemic

Turkey experienced some difficulties at the onset of the pandemic, such as ensuring PPEs for health workers and the general public, obtaining some more bedside ventilators to be ready for possible patient influx, preparing the pile of possible drugs for patients' treatment, etc. It may also be problematic to implement some of the CSAB decisions related to other ministries' roles and responsibilities.

To cover domestic needs, a transient ban on exporting PPEs and medical equipment is implemented initially. All industrial corporations are promoted to change their production lines to produce medical equipment especially ventilators. Domestic pharmaceutical companies are supported to produce meds to be used for the treatment of COVID-19 patients. However, there are more to be done to face pandemics, which is beyond the Ministry of Health's mandate.

With the strong support and commitment by the President, all line ministries worked in harmony to implement and put CSAB recommendations into practice. Border and airport controls, regulations on importing goods from risky areas, quickly initiating distance learning models for continuing education online, curfew practices, etc. are put into practice in an extraordinary manner.

## Arrival of SARS-COV-2 in Turkey: Public Health Measures and Multisectoral Activities Based on Whole-of-Government Approach

### The First SARS-CoV-2 Case

**Figure 1:**
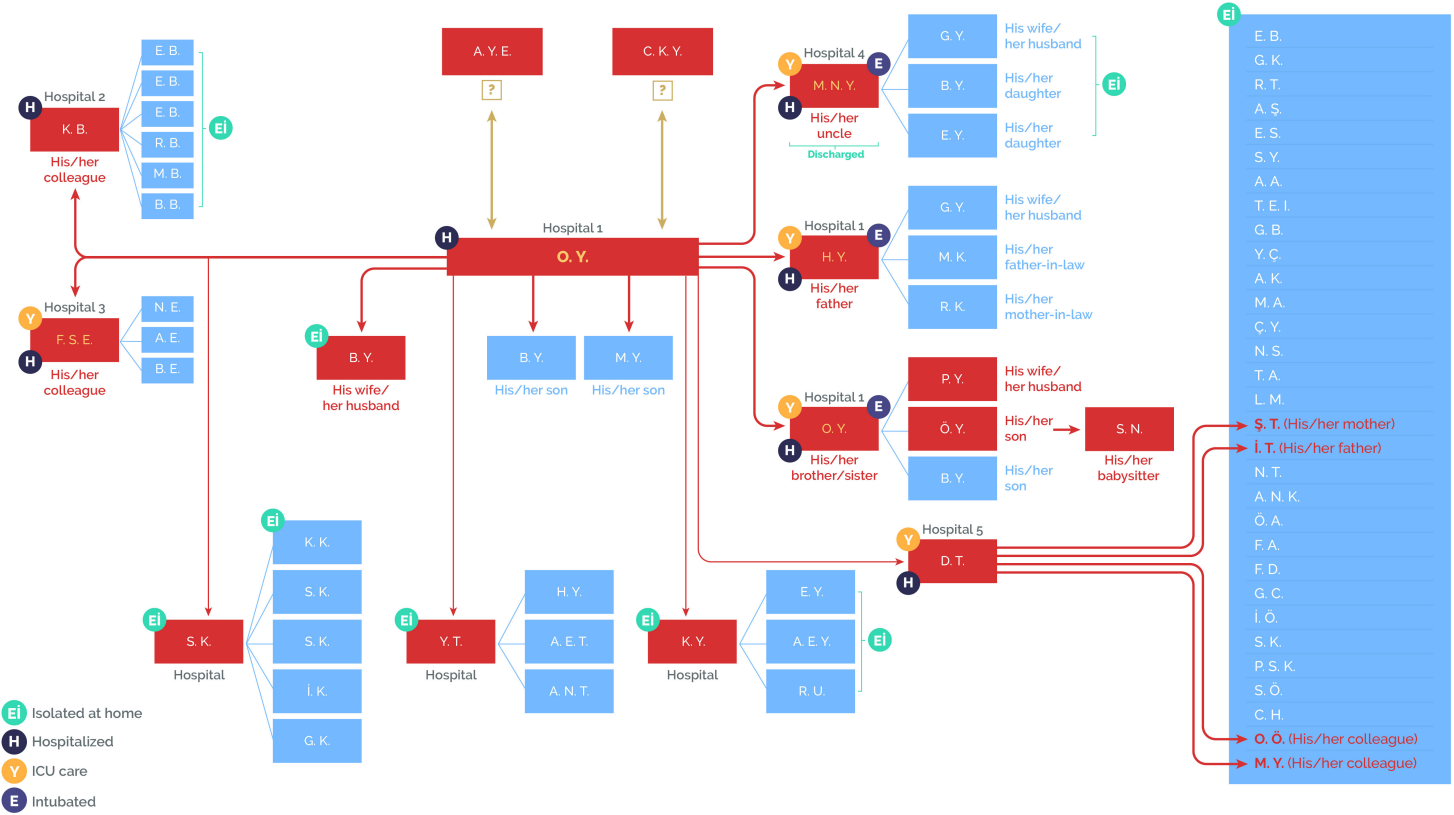
Contact-tracing scheme of the first case in Turkey. *Source:* Daily reporting from the Directorate General for Health Information Systems, Ministry of Health, Turkey (https://covid19.saglik.gov.tr/?_Dil=2).

Turkey announced the first confirmed case on 11 March, incidentally the same day that the WHO announced SARS-CoV-2 outbreak as a pandemic (14). Minister Koca later shared a graph showing how contact tracing of the first case was performed (Figure 1).

The day after the first case was reported, President Erdoğan led a ministerial cabinet meeting to initiate implementation of the response road map of the Turkish Government.

### Mitigation Measures

i Closure of On-Site Instruction in Education Institutions Across the Country

All primary, middle, and high schools and universities in Turkey were closed, effective the following Monday (16 March 2020), and online and TV broadcasting supported education for primary, middle, and high schools starting after a 1-week period of midterm break (15).

ii Banning of Mass-Gathering Events

A large number of measures to prevent mass gatherings were put into practice (16). All sport games; scientific, cultural, or artistic meetings; conferences; and congresses were postponed until further notice. Mosques and all places of worship, libraries, cafes, gyms, movie theaters, etc. were closed. Public banks started delivering pensions to retirees above the age of 76 years to their homes to help them stay at home.

iii Restrictive Measures for Public Officials

Public officials over 60 years of age and those suffering from chronic conditions (presumed to be at high risk of SARS-CoV-2 based on global evidence) were granted administrative leave (17, 18). Public institutions and organizations were ordered to allow alternating and flexible working schedules and enforce remote/tele-working if and where possible.

iv Additional Travel Restrictions

Flight bans were later extended to include most of the European countries.

v Curfews and Lockdowns

1. *Selective Curfew for the Elderly Over 65 Years and Establishment of “Vefa (Fidelity) Social Support Groups”* (18)

**Figure 2:**
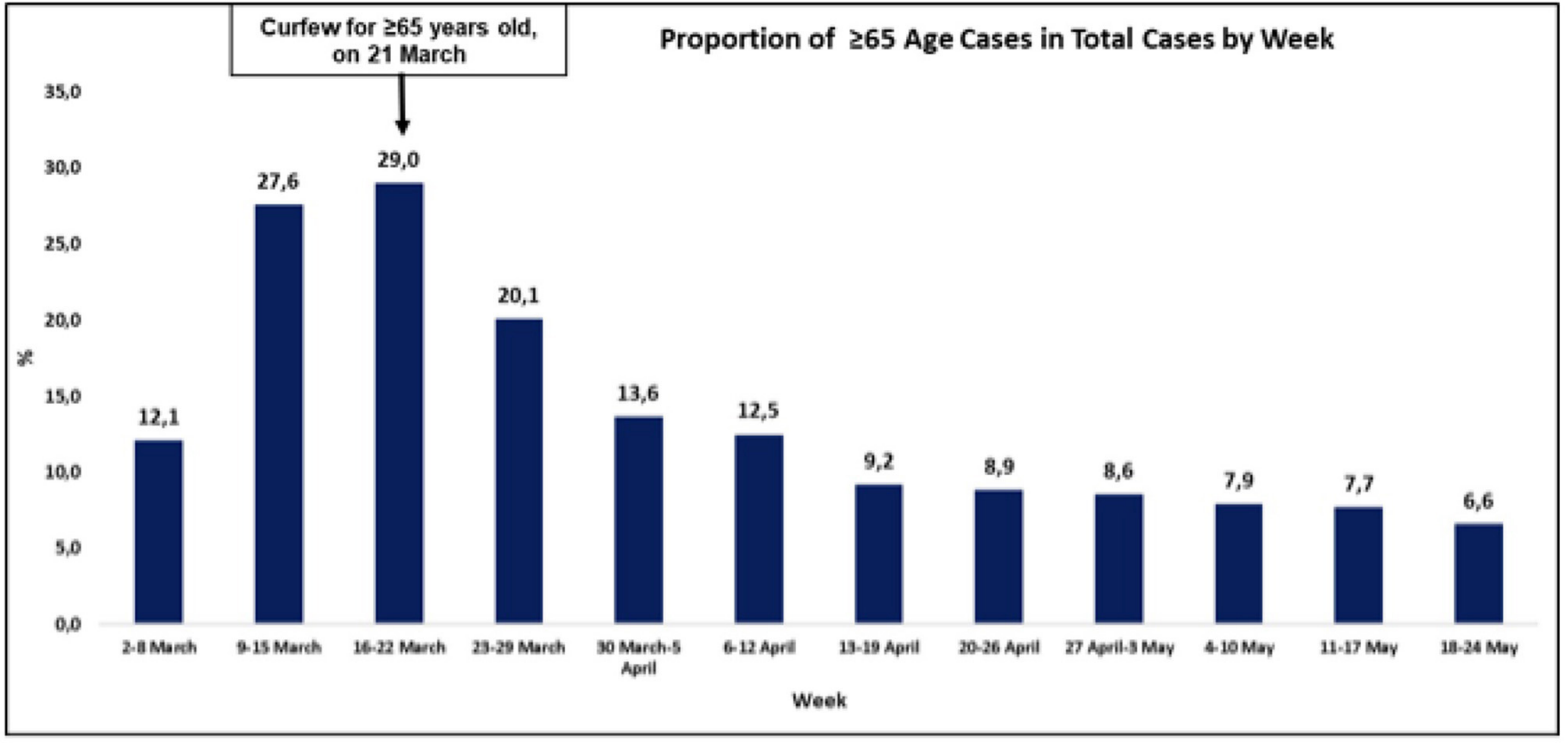
The incidence of new cases of SARS-CoV-2 in the elderly. *Source:* Daily reporting from the Directorate General for Health Information Systems, Ministry of Health, Turkey (https://covid19.saglik.gov.tr/?_Dil=2).

Effective from 22 March, a curfew was imposed for those over 65 years of age while their daily needs were met through newly established special teams called “Vefa (fidelity) social support groups.” These curfew measures for the elderly seemed to have played a major role in reducing the incidence of new cases of SARS-CoV-2 in the elderly (Figure 2).

2. *Curfew for Those Under 20 Years*

Ten days after imposing the curfew for the elderly over 65 years, curfew imposition was extended for those under age of 20.

vi Weekend Total Lockdowns

The curfew for the whole population was first imposed on the weekend of 11 April and continued till June in selected cities. It was extended to cover public holidays adjoining weekends.

vii In-Country Travel Restrictions

On 3 April, entrance ban to 30 metropolitan municipalities and provinces was announced by the President. All these measures are implemented with a whole-of-government approach, with active participation and contribution of all relevant authorities.

viii Economic Relief

An economic relief package of 100 billion TL (roughly 15 billion dollars) was announced by President Erdoğan on 18 March 2020 to address immediate financial woes of companies and costs in low-income households (15). With this package, the government also agreed to postpone tax liabilities, social security premium payments, and credit debts of employers in sectors worst affected by the crisis. The government also coordinated cash-raising campaigns and transferred 11.5 billion TL to families in need (2 billion TL was raised through an aid campaign called “We Are Enough For Each Other Turkey”), among other measures.

ix Incentives for Healthcare Workers

Special economic incentives for HCWs were provided by the government. Additional remuneration was granted to HCWs with a regulation published on 14 March 2020 (19). GSM operators in the country also provided 15 GB Internet packages for HCWs free of charge to facilitate continued contact of HCWs with their patients under isolation/quarantine and contact of HCWs with their own as well as patients' families and loved ones. Similarly, for those HCWs who could not commute or did not want to go home after their shifts for fear of transmitting the virus to family members/loved ones, alternate accommodations were provided free of charge.

x Free Health Coverage for SARS-CoV-2 for All

With a presidential decree published in the Official Gazette on 14 April 2020, all costs related to diagnosis and provision of medical treatment of persons with SARS-CoV-2 were made free of charge for all citizens and residents of Turkey (20).

xi Mental and Psychosocial Health

Some professionals working in healthy living centers and in some hospitals were trained and organized to provide psychosocial and mental health support to the community by placing such staff in at least one healthcare facility in each province.

xii Smartphone Apps/IT Usage

A specific software module for COVID-19 was added to the Public Health Management System software to ease surveillance of the disease and contact tracing.

A mobile application called Mental Health Support System was developed by the MoH to provide a direct channel between mental health professionals and HCWs, to protect the mental health and support the well-being of HCWs providing health care under challenging circumstances.

Another mobile application called “Hayat Eve Siğar” (Life Fits in Home) was also developed by the MoH, to inform, guide, and protect the public about areas with high exposure risk and by alerting them about high-risk behaviors. Residents could obtain a code through this application if that individual's travel between provinces was not restricted (not during the isolation period or recovery phase). Ten million residents have downloaded this application.

Special arrangements were also made to reduce the need for visits to healthcare facilities for purposes other than medical consultation, to reduce potential exposure risks for visitors as well as HCWs. Such measures included prescription refills for chronic diseases directly from pharmacies without a fresh prescription from a clinician.

xiii Role of Family Physicians

Family physicians have played a critical role in this response. They shouldered the provision of medical care in hospitals on the one hand and provided follow-up for vulnerable groups, such as the elderly, pregnant women and children, and refugees, on the other hand. They provided daily health checks of such vulnerable members of the community who were confined/isolated in their respective homes because of known close contact with confirmed SARS-CoV-2 patients but were asymptomatic and thus not hospitalized.

xiv Research and Development

1. *Clinical Trials for Vaccine Development*

The MoH has organized a committee to synchronize and coordinate all clinical trials related to SARS-CoV-2. Data from multicentric scientific trials are intended to be submitted for peer review and publication in various journals. Multiple institutions initiated research on vaccine development, therapeutics, and plasma convalescent therapy.

2. *Transfer and Sharing of Global/Regional Knowledge*

MoH officials organized several videoconferences with many countries and with three levels of WHO and other international organizations to acquire/share/transfer knowledge, emerging best practices, and experience gained.

## Pandemic Course in Turkey and Containment Measures

### The Course of the Pandemic in Turkey

**Figure 3:**
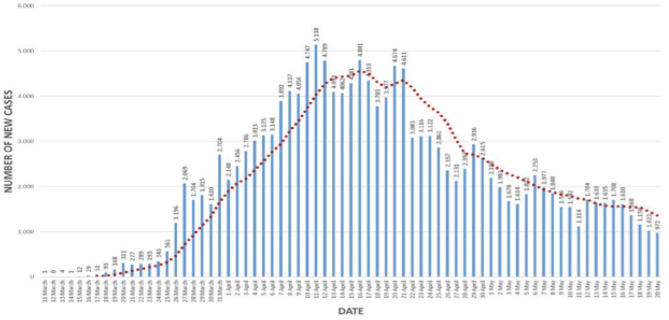
The epidemiological curve of SARS-CoV-2 cases reported in Turkey. *Source:* Daily reporting from the Directorate General for Health Information Systems, Ministry of Health, Turkey (https://covid19.saglik.gov.tr/?_Dil=2).

Containment measures in Turkey basically comprised four essential strategies: testing, (contact) tracing, treatment, and quarantine/isolation. The epidemiological curve of cases with SARS-CoV-2 reported in Turkey is displayed in Figure 3.

**Figure 4:**
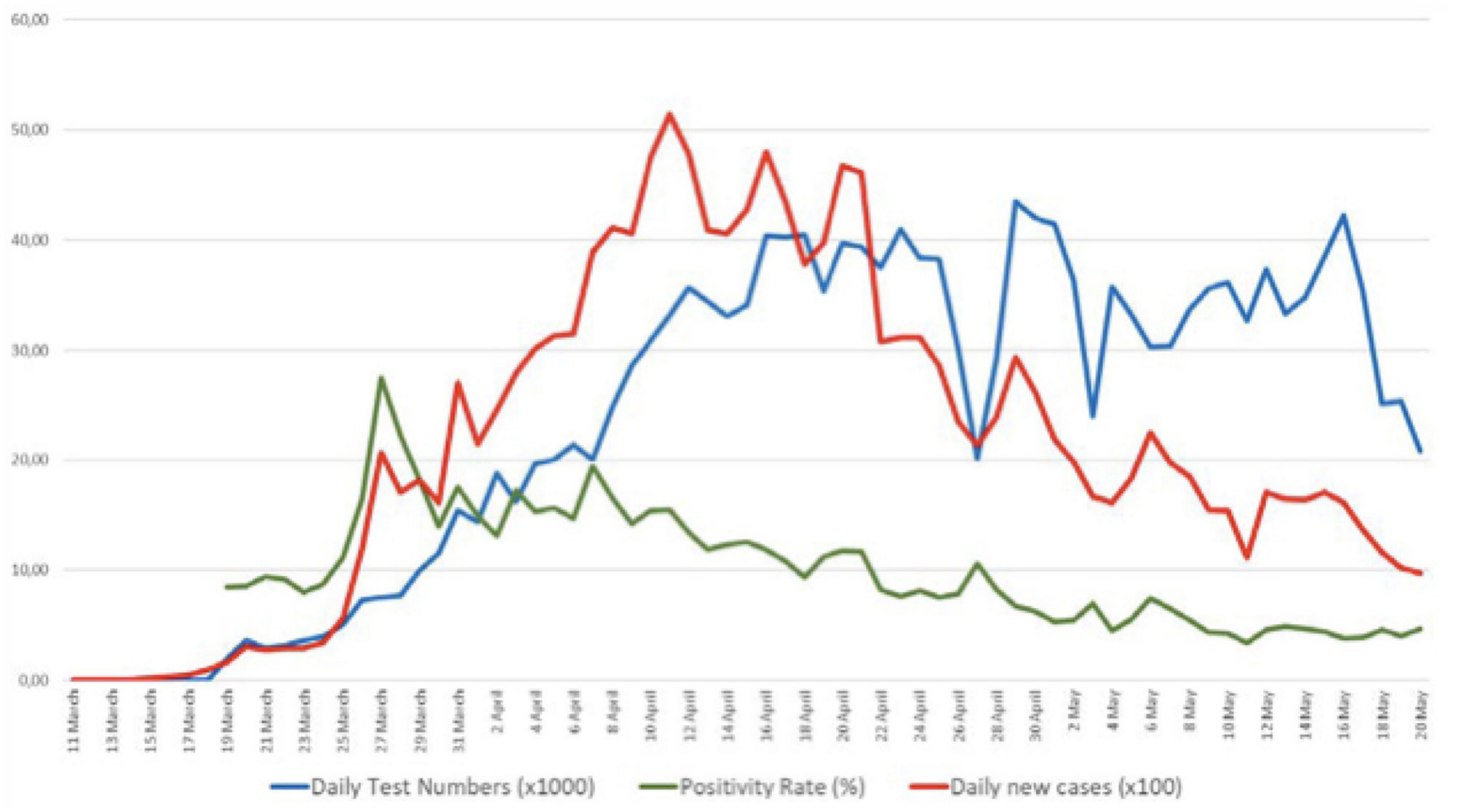
Testing capacity, positivity rate, and case numbers per 1,000 population starting from 11 March to 20 May. *Source:* Daily reporting from the Directorate General for Health Information Systems, Ministry of Health, Turkey (https://covid19.saglik.gov.tr/?_Dil=2).

The highest number of daily new cases was reported on 11 April with 5,138 cases, the peak of the pandemic in the current wave. The peak tapered to a daily new case of less than a thousand by 20 May 2020 (Figure 3). Another key attribute responsible for Turkey's successful course is its strong testing capacity; Turkey rapidly increased its daily testing capacity up to 40,000–50,000/day, one of the best in the region, while many other developed economies continued to face the testing glut. With its high testing capacity, Turkey was able to test for cases and rapidly trace and test close contacts early. It was also able to isolate/quarantine and/or treat cases and thus interrupt the transmission chains early and effectively, preventing the spread of the virus to new susceptible individuals (Figure 4).

All confirmed cases can access case management and treatment easily and free of charge. Treatment recommendations are given in the COVID-19 Guidelines developed by the CSAB and are updated regularly in line with new evidence and information.

### Testing, Contact Tracing, and Case Finding

Turkey has implemented a comprehensive contact-tracing strategy. More than 6,000 field teams, composed of three staff each, were organized all around the country for contact tracing and epidemiological investigation of cases that had interactions with confirmed cases. A special software called FITAS (Filiation, Isolation and Tracing System) has been prepared and used to monitor all tracking activities and to reach all contacts, family relatives, colleagues at work, and others. This application is used also for monitoring individuals isolated at their homes. Family physicians regularly check their health status and refer them to hospitals at the earliest stage if any symptom arises. Through this process, Turkey has been able to reach 99.6% of all contacts, that is, approximately 792,000 people (more than five persons per one confirmed case), and each contact was detected within a timeframe of <32 h (21).

**Figure 5:**
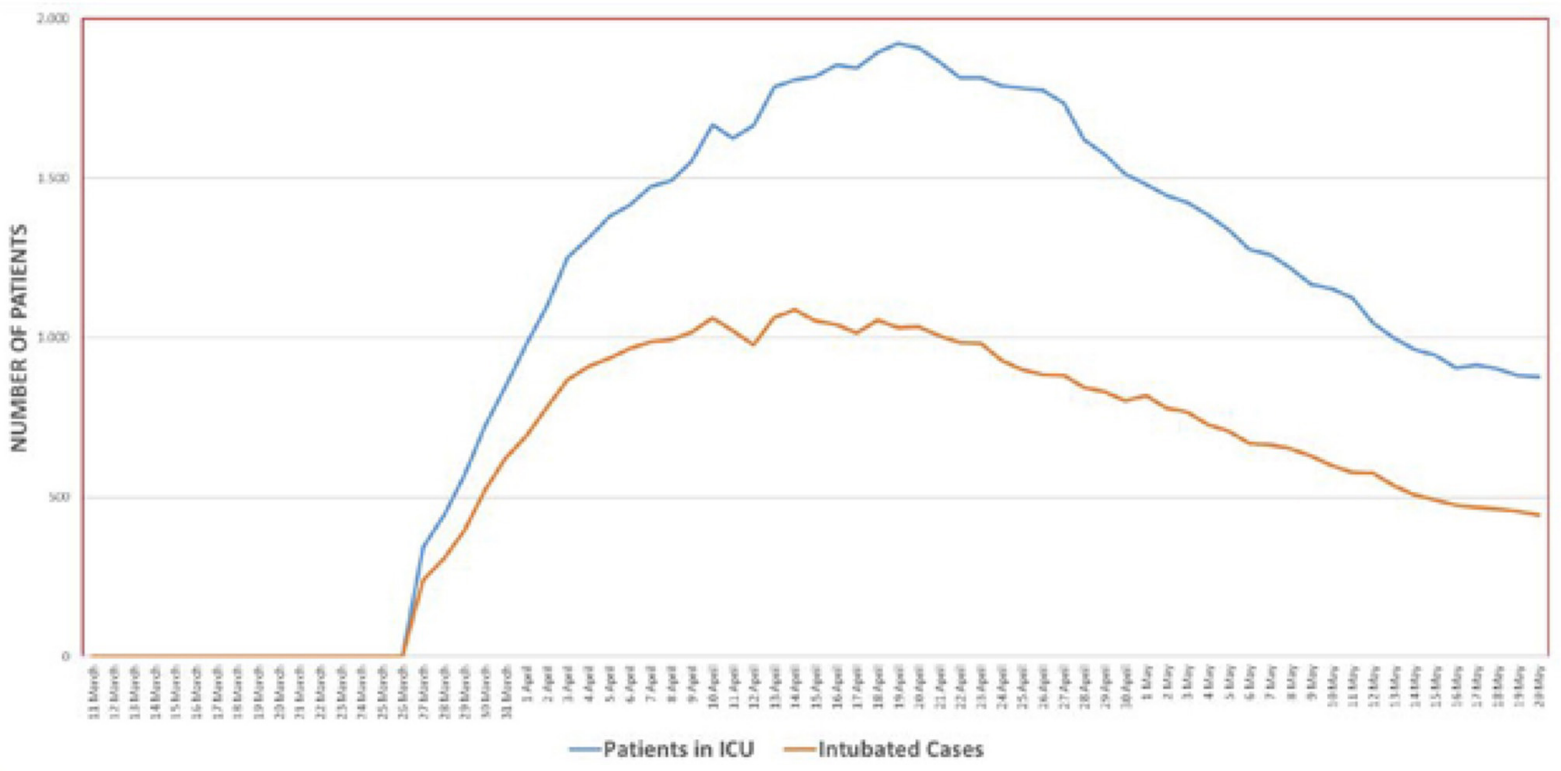
The number of patients in intensive care and those who were intubated. *Source:* Daily reporting from the Directorate General for Health Information Systems, Ministry of Health, Turkey (https://covid19.saglik.gov.tr/?_Dil=2).

While the testing capacity has averaged on or around 25,000/day or so, the yield rate of positive tests has gradually lowered from a high of 15% in mid-April to single digits (5–6%) by mid-May. This sustained lower yield despite the high number of tests underscores the fact that the country is now ready to implement cascading, controlled relaxation of lockdowns. Both the numbers of patients in need of ICU care and those in need to be intubated have decreased over time (Figure 5).

This could be attributed to the introduction of specific successful treatment protocols, as recommended by the CSAB. The Ministry of Health had recommended starting hydroxychloroquine and/or azithromycin treatment if the likelihood of pneumonia was high. The large number of cases with only mild pneumonia in hospital admissions also lowered the need for intensive care and intubation. The full efficacy and safety of such treatment regimens still remain to be ascertained after compilation and analysis of observational data supplanted with retrospective chart reviews of patients. Initial therapy regimen with hydroxychloroquine was elaborated recently by the CSAB, and they advised to give the drug only to hospitalized patients.

It may also be useful to compile additional evidence to document the benefits of early treatment with favipiravir. Additionally, the accumulated clinical evidence on what is beneficial ranges from high-flow oxygenation, nursing in the prone position, late intubation, and the use of immunomodulators (such as anakinra and tocilizumab) and anticoagulants as supportive therapies for case management, with improved health outcomes in severe cases. Early diagnosis, contact tracing, and case management have helped greatly by lowering new infections, improving prognosis, and reducing the strain on the healthcare system. The proportion of intubated patients compared with those in intensive care has remained stable (~50%) over time, also a proxy validation of effective case management practices (Figure 5).

## Leaving No One Behind

Turkey hosts 3.6 million Syrians and additional nearly one and a half million regular and irregular migrants within its borders. Only 60,000 Syrians are living in the camps, while the rest live within host communities scattered across various provinces. Although it would be relatively easier to provide health services to displaced populations living in camp settings, since >97% of Syrians in Turkey reside in host communities, Turkey established a network of migrant health centers for provision of health services and built these centers especially in provinces heavily inhabited by Syrians. In 2019, more than 17 million visits were recorded to these health facilities. Relevant health education and communication materials, including those for the pandemic, were developed in Arabic and English to facilitate linguistic and cultural acceptability by the refugee/migrant community. These migrant health centers helped to educate and train populations about the disease and the health protection measures they should take, and facilitated contact-tracing activities, especially for migrants. All related diagnostics and treatment procedures for SARS-CoV-2 during the pandemic are considered as emergencies, and accordingly, under this emergency approach, all services related to pandemic prevention and treatment were provided free of charge for both regular and irregular migrants as is the case with citizens (4).

Special arrangements were made to protect virus transmission in prisons and care homes for the elderly. Fixed staff teams, prescreened and determined to be non-infected, were arranged to work for longer hours in these facilities. Cases in prisons and care homes were isolated immediately in hospitals, and contacts of these cases were also screened and quarantined/isolated as needed. Meetings, visits, and transfers of prisoners were also postponed, minimizing exposures.

## Turkey's Contribution to International Solidarity

As an eminent and dutiful country as part of the international community, Turkey has been determined to fight an effective battle against COVID-19 at the national, regional, and global levels. Turkey's holistic approach, its deep-rooted state traditions, strong organizational structure, solidarity between the strong state and the nation, and effective leadership with political will—all such elements came to the fore, arming the nation in this fight.

Since the beginning of the outbreak, 136 countries and 8 international organizations have requested to cooperate with Turkey as part of their COVID-19 response efforts.

These cooperation requests were about:

- Sharing scientific and technical knowledge and experiences
- Donation of medication, medical supplies, and medical devices
- Provision of sales and export licenses for medication, medical supplies, and medical devices.

Under the coordination of the Presidency, the Ministry of Health, Ministry of National Defense, Ministry of Foreign Affairs, Turkish Red Crescent, Turkish Coordination and Cooperation Agency (TIKA), and Disaster and Emergency Management Presidency of Turkey (AFAD), as well as many professional organizations, civil society organizations, and international foundations and associations, took part in these aid efforts.

Turkey has supported more than 80 countries with personal protective equipment, diagnostic kits, medical devices, and medication and issued exceptional export licenses for 65 different countries and international organizations allowing the export of medication, personal protective equipment, and medical devices from Turkey to these end-beneficiaries.

In terms of scientific and technical knowledge and exchange of experiences, the Minister of Health, Dr. Fahrettin Koca, has conducted bilateral meetings with the Director-General of WHO, Regional Director of WHO European Region, and the ministers of health of the USA, Azerbaijan, the UK, Spain, Bulgaria, Libya, Pakistan, Romania, Tunisia, Kazakhstan, Russia, and Iran, as well as multilateral meetings at the Turkic Council. In addition, the CSAB members have taken part in meetings with the scientific committees of other countries.

Turkey has also tried to fulfill requests for personal protective equipment and ventilators from outside as much as possible while taking into consideration priority domestic needs. Turkey has continued to support cross-border healthcare services at the border with northern Syria during this period.

For Turkey, global cooperation and solidarity are part and parcel of COVID-19 response efforts. Accordingly, Dr. Fahrettin Koca's recommendations on the establishment of a “Supply Chain Group,” “Health Scientific Board,” and “Health Business Forum” were unanimously accepted by Member States of the Turkic Council, again setting an example among international platforms.

The words of Mevlana Celaleddin-i Rumi (famous Anatolian mystic philosopher), displayed on the boxes of supplies sent to other countries, constitute the essence of Turkey's efforts:

”*There is hope after despair and many suns after darkness.”*

Through the act of global solidarity exhibited in COVID-19 response efforts, Turkey has once again demonstrated that it may not be the wealthiest, but it is one of the most generous of countries.

## Toward the New Normal; Controlled Social Life

President Erdoğan announced a road map for normalization on 3 May. It is called “a new normal,” meaning some public health measures will be implemented permanently even after all facilities reopened. The CSAB provides advice on this normalization plan and prepares guidelines for various sectors. A research study is planned to determine the immunity level of the community at the provincial level; 150,000 samples will be collected for testing during that study.

Turkey's normalization is a dynamic process. Depending on up-to-date developments, some measures may be loosened earlier or later. The future of normalization will be decided not only by the impact of measures but also by public behavior. Therefore, public engagement plays a vital role. That is why the public is informed on a daily basis and communities are engaged efficiently throughout the process with a whole-of-society approach.

As also echoed by the WHO, countries need to ensure that they have capacities in place to detect and manage any upsurge in the number of cases once the transition period to a new normal is initiated. Despite low levels of intensive care and hospital bed occupancy ratios, construction of some additional pandemic hospitals and development of health system capacity is ongoing in Turkey.

## Conclusions

Turkey has successfully turned the corner in the current wave of the pandemic and stands among the countries with lower mortality rates generally but remarkably low mortality rates in the elderly. A multitude of factors seem to have worked in tandem and may hold the answers to these results:

1. Political commitment and leadership, multisectorial engagement, and whole-of-government approaches are all pillars of any public health struggle, as emphasized by the WHO and demonstrated by Turkey's experience.
2. The proportion of elderly (>65 years) in the overall population of Turkey is smaller as compared with countries with higher deaths rates (e.g., 6.5% as compared with >14% in Italy). Specific preventive and testing measures were instituted early to protect the elderly and prevent the spread of infection.
3. Early imposition of selective curfew for the elderly and people with chronic conditions protected them from being exposed to the circulating virus.
4. Turkey was an avid observer and a quick learner to adopt selective containment and mitigation measures closely observing the experiences of other countries and applying these nationally. Turkey adapted its national guidelines several times according to the latest knowledge and best practices—*Think globally and apply locally*.
5. Turkish SARS-CoV-2 guidelines were prepared with clear evidence-based recommendations, providing standardized therapeutic algorithms to all stakeholders around the country.
6. Strategic stockpiles, local production, and prepositioning allowed Turkey to avoid any critical shortages of personal protective equipment, drugs, and medical equipment.
7. Pre-pandemic high ICU bed/population ratios allowed the dilution of the strain on critical care systems even at the peak of the pandemic, and the highest occupancy for ICU beds did not exceed 60%.
8. Turkey has been active and flexible to extend the use of all possible treatment options to clinicians, ranging from antivirals to even some of the regimens from traditional Chinese medicine, and updated its guidelines according to clinical experiences.
9. Hydroxychloroquine is given to all positive and suspected cases as soon as diagnosis of SARS-CoV-2 is made. Early medication particularly with antivirals and high-flow oxygen seems to play some protective role, precluding the need for use of mechanical ventilators in the ICU.
10. Late intubation and prone position seem to have contributed to improved health outcomes in patients in the ICU.

Turkey is not among the richest of the countries. In fact, Turkey is the 17th country by virtue of population size and the 19th largest economy. Its gross domestic product (GDP)/capita scale [as measured by the purchasing power parity (PPP)] is around 75th in the world. The total expenditure on health is not more than 5% of GDP and that means a little more than $1,000 is spent on health per capita (on a PPP scale). Despite this, Turkey has been the most generous country as measured by its GDP. Turkey continued to support global solidarity and unity by sending lifesaving personal protective equipment and other supplies to countries in need without creating any shortages of these much-needed products at home.

Turkey's experience with its therapeutic algorithms, political and policy decisions, and public health measures have kept mortality rates from COVID-19 low particularly among the elderly. With technical backstopping from the WHO and other stakeholders, Turkey offers lessons and best practices that could be useful in contributing to the global health arsenal against the pandemic.

## The Way Forward

With all critical indicators of the severity of the pandemic tapering consistently and continually for over 4 weeks, since mid-April, Turkey is preparing for a measured exit into a socially controlled life starting from June 2020. Nonetheless, it will be extremely critical for communities to ensure compliance with social distancing, personal hygiene, and personal responsibilities to keep the infection rates low and the spread of the disease in check to ensure that there is no second peak in the initial wave that the country has successfully tamed. A critical marker is the looming autumn (fall) season, which is the flu season. Social distancing and personal hygiene coupled with compliance with flu vaccination are the interventions that can see Turkey safely through the fall.

Turkey will also continue to work with the WHO to ensure the containment, mitigation, and therapeutic and case management measures that have worked to its unique advantage in turning the corner, and best practices are shared and applied in a timely manner for the ultimate benefit of humanity (22).

## Data Availability Statement

The data analyzed in this study is subject to the following licenses/restrictions: **Datasets belong to Ministry of Health**. Requests to access these datasets should be directed to Emine Alp Mese, eminealpmese@gmail.com.

## Author Contributions

BK wrote the manuscript. IS, AT, PU, AM, and EM read and edited the manuscript. All authors contributed to the article and approved the submitted version.

## Conflict of Interest

The authors declare that the research was conducted in the absence of any commercial or financial relationships that could be construed as a potential conflict of interest.

## Footnotes

^1^The number of ICU beds was 2,214 in 2002, 869 of which were in public hospitals. As of 2019, the total number of total ICU beds in Turkey is 39,279, including 16,887 in public hospitals.

^2^GDOs are defined as entities that constitute a fully integrated part of the WHO Regional Office for Europe and its programs but which are physically located outside Copenhagen.

^3^Communiqué on Notification and Reporting System of Communicable Diseases (& November 2004). Official Gazette no: 25635 (in Turkish).

^4^Regulation on Surveillance and Control of Communicable Diseases (30 May 2007). Official Gazette no: 26537. Updated on May 4th 2019. Official Gazette No: 30764 (in Turkish).
